# Genome Sequence of Mycobacteriophage Momo

**DOI:** 10.1128/genomeA.00601-15

**Published:** 2015-06-18

**Authors:** Welkin H. Pope, Elizabeth A. Bina, Indraneel S. Brahme, Amy B. Hill, Philip H. Himmelstein, Sara M. Hunsicker, Amanda R. Ish, Tinh S. Le, Mary M. Martin, Catherine N. Moscinski, Sameer A. Shetty, Tomasz Swierzewski, Varun B. Iyengar, Hannah Kim, Claire E. Schafer, Sarah R. Grubb, Marcie H. Warner, Charles A. Bowman, Daniel A. Russell, Graham F. Hatfull

**Affiliations:** Department of Biological Sciences, University of Pittsburgh, Pittsburgh, Pennsylvania, USA

## Abstract

Momo is a newly discovered phage of *Mycobacterium smegmatis* mc^2^155. Momo has a double-stranded DNA genome 154,553 bp in length, with 233 predicted protein-encoding genes, 34 tRNA genes, and one transfer-messenger RNA (tmRNA) gene. Momo has a myoviral morphology and shares extensive nucleotide sequence similarity with subcluster C1 mycobacteriophages.

## GENOME ANNOUNCEMENT

Mycobacteriophages provide insights into viral diversity and evolution as well as facilitating development of tools for mycobacterial genetics (1). Exploration of viral diversity is facilitated by the Science Education Alliance Phage Hunters Advancing Genomics and Evolutionary Science (SEA-PHAGES) program, in which undergraduate students discover and characterize novel bacteriophages (2). Mycobacteriophage Momo was isolated by SEA-PHAGES students from a soil sample collected in Pittsburgh, Pennsylvania, by direct plating on *Mycobacterium smegmatis* mc^2^155*.* Momo forms clear circular plaques ranging from pinprick sized to 2.5 mm in diameter, and electron microscopy shows that is has a myoviral morphology.

Momo was purified and amplified, and its DNA was isolated and sequenced using an Illumina MiSeq with 140-bp single-end runs. Reads were assembled using Newbler to give a major contig of 154,533 bp with 182-fold coverage. The genome is circularly permuted and was bioinformatically linearized consistent with closely related genomes. The Momo genome has a 64.7% G+C content. A total of 233 protein-encoding Momo genes were predicted using Glimmer and GeneMark, using both heuristic and *M. smegmatis* models, followed by manual inspection and refinement. Functions were assigned to 40 of the genes using Phamerator (3), BLASTp, and HHPred, and functional assignments include virion structural proteins and DNA metabolism proteins including the alpha subunit of DNA polymerase III, RusA, RecA, and SSB. The large terminase subunit (gp255) is the only predicted gene product to contain an intein. Using Aragorn and tRNA-scan, 34 tRNA genes and one transfer-messenger RNA (tmRNA) gene were identified.

Comparison of Momo with other sequenced phages showed that it is closely related to mycobacteriophages in cluster C, which includes the generalized transducing phage Bxz1 (4, 5). The nearest relative is the subcluster C1 phage Ghost with which it shares 99% identity over a span of 99% of its genome length. However, there are two Ghost genes that are absent from Momo, and two genes present in Momo that are absent from Ghost. Each of these results from precise insertions or deletions of the reading frame without altering the flanking genes, and because of the close nucleotide similarity between these phages, must have resulted from relatively recent recombination events. However, none of the inserted or deleted genes are transposon, intron, or intein-related.

The cluster C phages have the largest repertoire of tRNA genes of any of the mycobacteriophages (6). The 35 predicted tRNAs encoded by Momo have anticodons indicating there are specificities for all 20 amino acids, corresponding to all codons sets with the exception of the 5′-TCN serine codons. Overall, the Momo codon usage is similar to that of its *M. smegmatis* host, and it is unclear what roles the tRNAs play in Momo and its cluster C relatives.

### Nucleotide sequence accession number. 

The Momo genome sequence is available from GenBank under the accession no. KR080196.
